# Novel Neuropathic Pain Mechanisms Associated With Allergic Inflammation

**DOI:** 10.3389/fneur.2019.01337

**Published:** 2019-12-17

**Authors:** Takayuki Fujii, Ryo Yamasaki, Jun-ichi Kira

**Affiliations:** Department of Neurology, Graduate School of Medical Sciences, Neurological Institute, Kyushu University, Fukuoka, Japan

**Keywords:** neuropathic pain, allergic inflammation, glia, endothelin, plexin D1

## Abstract

Allergic diseases are associated with central and peripheral nervous system diseases such as autism spectrum disorders and eosinophilic granulomatosis with polyangiitis, which frequently causes mononeuritis multiplex. Thus, it is possible that patients with an atopic constitution might develop multifocal inflammation in central and peripheral nervous system tissues. In a previous study in Japan, we reported a rare form of myelitis with persistent neuropathic pain (NeP) in patients with allergic disorders. However, the underlying mechanism of allergic inflammation-related NeP remains to be elucidated. First, we analyzed the effect of allergic inflammation on the nociceptive system in the spinal cord. Mice with atopy showed microglial and astroglial activation in the spinal cord and tactile allodynia. In a microarray analysis of isolated microglia from the spinal cord, endothelin receptor type B (EDNRB) was the most upregulated cell surface receptor in mice with atopy. Immunohistochemical analysis demonstrated EDNRB expression was upregulated in microglia and astroglia. The EDNRB antagonist BQ788 abolished glial activation and allodynia. These findings indicated that allergic inflammation induced widespread glial activation through the EDNRB pathway and NeP. Second, we investigated whether autoantibody-mediated pathogenesis underlies allergic inflammation-related NeP. We detected specific autoantibodies to small dorsal root ganglion (DRG) neurons and their nerve terminals in the dorsal horns of NeP patients with allergic disorders. An analysis of IgG subclasses revealed a predominance of IgG2. These autoantibodies were mostly colocalized with isolectin B4- and P2X3-positive unmyelinated C-fiber type small DRG neurons. By contrast, immunostaining for S100β, a myelinated DRG neuron marker, showed no colocalization with patient IgG. Immunoprecipitation and liquid chromatography-tandem mass spectrometry identified plexin D1 as a target autoantigen. Patients with anti-plexin D1 antibodies often present with burning pain and thermal hyperalgesia. Immunotherapies, including plasma exchange, are effective for NeP management. Therefore, anti-plexin D1 antibodies may be pathogenic for immune-mediated NeP, especially under allergic inflammation conditions. Thus, allergic inflammation may induce NeP through glial inflammation in the spinal cord and the anti-plexin D1 antibody-mediated impairment of small DRG neurons.

## Introduction

Allergic diseases are associated with central and peripheral nervous system diseases such as autism spectrum disorders (1–3) and eosinophilic granulomatosis with polyangiitis, which frequently causes mononeuritis multiplex (4, 5). These observations indicate that patients with an atopic constitution develop multifocal inflammation in central nervous system (CNS) and peripheral nervous system (PNS) tissues (6).

We previously reported a rare form of myelitis with persistent neuropathic pain (NeP) in Japanese patients with allergic diseases (7, 8). Nationwide surveys have found that this form of myelitis is widely distributed in Japan (6, 9). Similar cases have also been reported in Western countries (10, 11). Patients with this form of myelitis as well as atopy often showed cervical cord involvement, mainly in the posterior lesion, and exhibited sensory impairment including NeP in all four limbs (6, 9). We found a loss of myelin and axon and eosinophil infiltration in biopsied spinal cord lesions from these patients (12, 13). Thus, we designated this form of myelitis “atopic myelitis (AM)” and established diagnostic criteria (14). Definite AM is defined as: (1) patients meeting the absolute criteria [myelitis with unknown etiology; positivity for allergen-specific IgE; and absence of brain MRI lesions fulfilling the Barkhof criteria for MS (15)] plus the pathological criteria (spinal cord biopsy samples showing existence of perivascular lymphocyte cuffings with various degrees of eosinophil infiltration, sometimes accompanied by granuloma); or (2) patients meeting the absolute criteria plus at least two of the three supporting positive criteria [present or past history of atopic disease; serum hyperIgEemia; increased level of interleukin (IL)-9 or eotaxin in cerebrospinal fluid (CSF)] plus one supporting negative criterion (no oligoclonal bands in CSF). Probable cases of AM are defined as: (1) patients meeting the absolute criteria plus one of the supporting positive criteria plus the one supporting negative criterion; or (2) patients meeting the absolute criteria plus at least two of the supporting positive criteria. In patients with AM, there were significant positive correlations between disease duration and Kurtzke Expanded Disability Status Scale score (16) and Sensory Functional scale score (17). However, the underlying mechanism of allergic inflammation-related NeP remains to be elucidated.

Recent studies have established a crucial role of immune system activation in modulation of NeP (18, 19). Pro-inflammatory cytokines, such as tumor necrosis factor (TNF)-α, interferon gamma (IFNγ), IL-1β, IL-6, and IL-17, were shown to be elevated in sera and CSF of patients with NeP (20, 21). Because receptors for these cytokines are expressed on sensory neurons, pro-inflammatory cytokines may exert direct effects on nociceptive sensory neurons and induce NeP. Moreover, treatment with anti-inflammatory cytokines, such as IL-4 and IL-10, was reported to alleviate NeP in animal models (22, 23). Moreover, passive transfer of Th1 cells to athymic nude rats lacking mature T cells enhanced pain hypersensitivity in the recipient mice (24). In contrast, passive transfer of polarized Th2 cells attenuated pain hypersensitivity in the recipient mice. These findings suggest that Th2-dominant allergic inflammation may be protective for NeP. However, in clinical practice, we often encounter patients with both allergic disease and severe NeP (6), suggesting that other NeP mechanisms are operative. Accumulating evidence indicates that activation of spinal microglia, resident macrophages in the CNS, is crucial for NeP generation and modulation (25, 26). Peripheral nerve damage induces microglial activation in the dorsal horn of the spinal cord. Activated microglial mediators in the spinal dorsal horn, such as TNF-α, IL-1β, and brain-derived neurotrophic factor (BDNF), increase excitatory synaptic transmission and cause NeP via neuron-glial interactions (27). We further focused on B cell hyperactivation, which induces NeP through production of autoantibodies against antigens in the somatosensory pathway in response to the allergic condition (19, 28). Indeed, autoantibodies against sensory neurons were detected in autoimmune diseases associated with pain, such as Guillain–Barré syndrome (29) and complex regional pain syndrome (CRPS) (30), and depletion of B cells reduced NeP in CRPS model mice (31).

In this Mini Review, we will discuss the possible NeP mechanisms associated with allergic inflammation, on the basis of findings from animal models of allergic disease and autoantibodies against sensory neurons of patients with allergic diseases.

## Allergic Inflammation Induces Neuropathic Pain Through The Activation Of Glial Cells

First, we analyzed the effect of allergic inflammation on the nociceptive system of the spinal cord in an animal model of allergic disease (32). We induced atopic diathesis, bronchial asthma, or atopic dermatitis in C57BL/6 mice by intraperitoneal sensitization with ovalbumin (OVA) (50 μg) and aluminum hydroxide hydrate (2 mg) on days 0, 7, and 14 (atopic diathesis model), followed by nasal aspiration of OVA solution (2.5 mg/ml) for 4 consecutive days (days 15–18) (bronchial asthma model) or direct OVA application (100 μg) on tape-stripped skin (atopic dermatitis model). Mice with atopy showed microglial and astroglial activation in the dorsal horn of the spinal cord. A higher expression of FBJ murine osteosarcoma viral oncogene homolog B (FosB), a neuronal activation marker, was also seen in the dorsal horn of mice with atopy compared with mice without atopy. Additionally, we found activated endothelial cells and extravasation of serum albumin in atopic mice, suggesting blood–brain barrier (BBB) impairment. There was neither demyelination nor axonal degeneration in the spinal cord of mice with atopy. We used von Frey filaments to evaluate tactile allodynia in mice with atopy (33) and found that atopy model mice had severe tactile allodynia.

In a microarray analysis of isolated microglia from the spinal cord of mice with atopy, microglia showed an augmented pro-inflammatory signature, including IL-1β, CD38, and prostaglandin-endoperoxide synthase 2, which are known to be upregulated in activated microglia (34, 35). Endothelin receptor type B (EDNRB) was the most upregulated cell surface microglial receptor in mice with atopy. Immunohistochemical analysis confirmed that EDNRB expression was upregulated in microglia and astroglia, and that spinal cord neurons did not express EDNRB. Meanwhile, endothelin receptor type A (EDNRA), another main receptor for endothelin, was not detected in microglia, astroglia, and neurons of the spinal cord of atopic mice. We further found increased levels of endothelin-1 (ET-1), an EDNRB ligand, in serum by ELISA, and observed marked up-regulation of ET-1 in alveolar epithelial cells and epidermis of atopic mice by immunohistochemistry. We then analyzed whether a selective EDNRB antagonist, BQ-788, would affect glial activation and tactile allodynia in atopic mice. BQ-788 treatment abolished microglial, astroglial, and neuronal activation and allodynia. Because the neuronal expression of EDNRB was not detected in atopic mice, the EDNRB antagonist primarily acted on microglia and astroglia rather than neurons. Thus, microglia and astroglia are important for the emergence of allergic inflammation-related NeP via the ET-1/EDNRB pathway.

We also conducted a neuropathological examination of autopsied spinal cord lesions from a patient with AM. We found microglial and astroglial activation in the dorsal horn of the spinal cord and the loss of myelin and axons, as seen in previously biopsied AM cases (12, 13). EDNRB expression was upregulated in microglia and astroglia, similar to in our atopy model mice. Moreover, we found elevated serum ET-1 levels in AM patients compared with healthy controls without atopy. Together, these findings indicate that allergic inflammation induces widespread glial activation, which persistently activates the nociceptive system in the spinal cord via the ET-1/EDNRB pathway.

## Anti-Plexin D1 Antibody-Related Neuropathic Pain In Patients With Allergic Diseases

Allergic inflammation can enhance autoantibody production (28) and plasma exchange has been reported to improve NeP in patients with AM (6, 36). Therefore, we investigated whether an autoantibody-mediated mechanism underlies allergic inflammation-related NeP.

We screened novel autoantibodies against dorsal root ganglion (DRG) neurons and the dorsal horn, which are involved in generating NeP, in patients with various neurologic diseases including AM, using a tissue-based indirect immunofluorescence assay (IFA) (37). We found specific autoantibodies against small DRG neurons and their nerve terminals in the dorsal horn of the spinal cord (37), and these autoantibodies were more frequently detected in patients with NeP than subjects without NeP (10% vs. 0%; *p* < 0.05). IgG subclass analysis revealed a predominance of IgG2, which weakly activates complement. These autoantibodies mostly colocalized with isolectin B4 (IB4)- and P2X3-positive unmyelinated C-fiber type small DRG neurons. By contrast, immunostaining for S100β, a myelinated DRG neuron marker, showed no colocalization with patient IgG. These findings showed that NeP patients' IgG binding was restricted to unmyelinated DRG neurons. In the dorsal horn of the spinal cord, patient IgG axonal staining colocalized with a lamina I marker calcitonin gene-related peptide (CGRP) and lamina II marker IB4. Therefore, IgG binding in patients with anti-small DRG neuron antibodies was restricted to the superficial dorsal horn (laminae I and II). These autoantibodies also bound to vasoactive intestinal peptide (VIP)-positive postganglionic parasympathetic nerve fibers in the skin. In western blotting (WB) using mouse DRG, these autoantibodies recognized a common 220 kDa band. Liquid chromatography-tandem mass spectrometry with immunoprecipitates revealed plexin D1 was the autoantigen.

Plexin D1 is a receptor for semaphorin 3E, an axon guidance factor and immune regulator (38) expressed in the nervous system, B cells, macrophages, endothelial cells, and skin (38). Given that the presence of plexin D1 in DRG sensory neurons has not been investigated, we assessed the expression of plexin D1 in human DRG sensory neurons (37). Immunohistochemical analysis of human DRG and spinal cord tissues with an anti-human plexin D1 antibody revealed that plexin D1 was expressed in small DRG neurons and the superficial dorsal horn. The immunostaining of small DRG neurons and spinal dorsal horn by IgG from all anti-small DRG neuron antibody-positive patients was removed by pre-incubation with recombinant human plexin D1 extracellular domain in a concentration-dependent manner (37). Therefore, we confirmed plexin D1 is a relevant autoantigen. Additionally, plexin D1 extracellular domain contains antigenic epitopes for autoantibody recognition. Then, we performed a propidium iodide (PI) assay to assess plasma membrane permeability using dissociated mouse DRG neurons and heat-inactivated sera from NeP patients with anti-plexin D1 antibodies. Heat-inactivated sera from NeP patients with anti-plexin D1 antibodies showed a significant increase in the percentage of PI-positive cells compared with those without anti-plexin D1 antibodies (37). These findings suggest that anti-plexin D1 IgG2 antibodies may invade the DRG where the BBB and blood–nerve barrier are absent, bind to plexin D1 on the surface of unmyelinated C-fiber type DRG neurons, and impair the plasma membranes of small pain-conveying neurons, resulting in their dysfunction.

In Table 1, we have summarized the clinical features of patients with anti-plexin D1 antibodies based on our previous study (37). The patients with anti-plexin D1 antibodies were predominantly female, although the difference in anti-plexin D1 antibody positivity rates between female and male patients with NeP was not significant (12.3 vs. 5.4%; *p* = 0.33). The age at onset was relatively young. The clinical courses were relapsing or fluctuating. The underlying neurological diseases of 11 patients with anti-plexin D1 antibodies included atopic myelitis, neuromyelitis optica spectrum disorders, multiple sclerosis, neurosarcoidosis, and erythromelalgia. The common comorbidities in patients with anti-plexin D1 antibodies were allergic diseases and collagen diseases. The patients commonly developed burning pain, thermal hyperalgesia, and peripheral vascular dysfunction symptoms. The current perception threshold test showed abnormalities of C-fibers. Plasma exchange and intravenous methylprednisolone pulse therapy were effective for NeP management. These findings suggest that anti-plexin D1 antibodies may be pathogenic in immune-mediated NeP, especially under allergic inflammation conditions.

**Table 1:** Clinical findings for 11 patients with anti-plexin D1 antibodies.

**Characteristic**	**Summary**
Female sex, number (%)	9 (81.8)
Age at onset, mean ± SD (range), years	26.3 ± 13.3 (12–53)
Underlying diseases, number (%)	AM 6 (54.5), NMOSD 2 (18.2), RRMS 1 (9.1), neurosarcoidosis 1 (9.1), erythromelalgia 1 (9.1)
Coexisting disorders, number (%)	Allergic diseases 10 (90.9), collagen-vascular diseases 4 (36.4), malignant neoplasms 1 (9.1)
Clinical course, number (%)	Relapsing 9 (81.8), fluctuating 2 (18.2)
Neurological manifestations, number (%)	NeP 11 (100), sensory impairment 11 (100), motor weakness 10 (90.9), hyperreflexia 10 (90.9), peripheral vascular autonomic dysfunction symptoms 5 (45.5), hand muscle atrophy 2 (18.2), visual impairment 2 (18.2)
Quality of NeP, number (%)	Burning 6 (54.5), tingling 6 (54.5), thermal hyperalgesia 5 (45.5), allodynia 2 (18.2), pinprick hyperalgesia 2 (18.2), squeezing 2 (18.2)
Electrophysiological findings, number (%)^a^	MEP abnormality of CNS 8 (72.7), CPT abnormality of C-fiber 6 (100), SEP abnormality of CNS 4 (36.4), SEP abnormality of PNS 3 (27.3), NCS abnormality 3 (33.3), QSART abnormality 1 (100)
Immunotherapy response for NeP, number (%)^b^	Improved 7 [mPSL pulse 4 and mPSL pulse plus PE 3] (100)

a *Percentage among tested patients who underwent each electrophysiological examination*.

b *Percentage among patients treated with various immunotherapies. CNS, central nervous system; CPT, current perception threshold; MEP, motor-evoked potentials; mPSL, methylprednisolone; NCS, nerve conduction study; NeP, neuropathic pain; NMOSD, neuromyelitis optica spectrum disorders; PE, plasma exchange; PNS, peripheral nervous system; QSART, quantitative sudomotor axon reflex test; RRMS, relapsing-remitting multiple sclerosis; SEP, somatosensory-evoked potentials*.

## Hypothetical Mechanisms Underlying Allergic Inflammation-Related Neuropathic Pain

### Glial Activation in Allergic Inflammation

Allergic diseases are associated with a risk for autism spectrum disorders (ASD) and attention-deficit and hyperactivity disorder (ADHD) (1, 2, 39). Moreover, microglia and autoantibodies against brain proteins are also associated with the pathogenesis of ASD (40–42). A recent transcriptome study using cortical tissue samples from patients with ASD showed microglial activation in cortical tissues of ASD patients (43). In an animal model of ASD, microglia from the offspring of mothers with allergic asthma exhibited epigenomic alterations in dysregulated genes (44). Therefore, allergic inflammation may contribute to the pathogenesis of ASD through microglial activation. However, it is unknown how allergic inflammation causes microglial activation. ASD children had significantly higher serum levels of anti-myelin basic protein (MBP) and anti-myelin-associated glycoprotein (MAG) antibodies than healthy children and the levels of autoantibodies against MBP and MAG were significantly correlated with the presence of allergic symptoms (45). Therefore, allergic inflammation might induce the production of autoantibodies against neurons and glial cells, which leads to CNS damage. However, no specific autoantibodies produced by allergic inflammation have been identified.

In our previous study (32), expression of EDNRB was upregulated in spinal microglia and astroglia from atopic mice and an autopsied AM case. By contrast, expression of EDNRA was not detected in microglia and astroglia of atopic mice. In the normal condition, expression of EDNRA in the spinal cord is observed in vascular smooth muscle cells and the superficial dorsal horn (primary afferent nerve fibers), while expression of EDNRB in the spinal cord is observed in radial glia, a small population of astroglia, ependymal cells, and vascular endothelial cells (46) (Supplementary Table 1). Therefore, allergic inflammation can induce overexpression of EDNRB in microglia and astroglia in the spinal cord.

**Figure 1:**
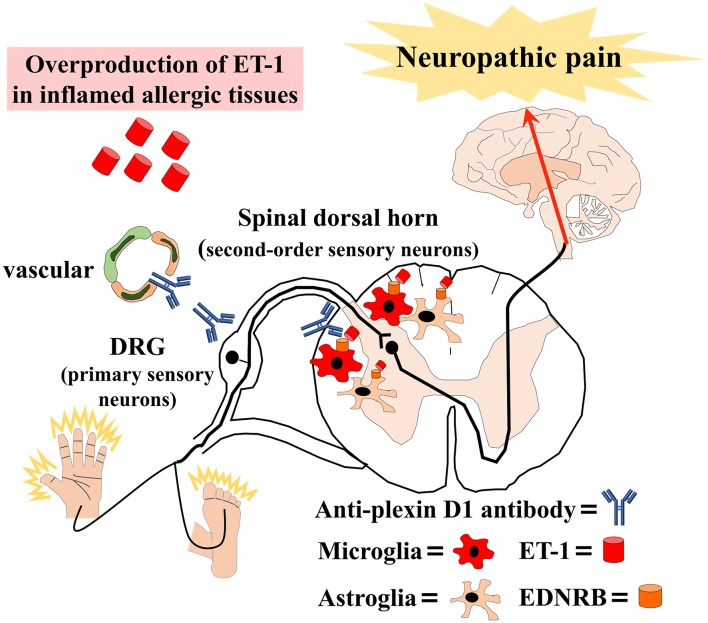
Schematic overview of our hypothesis that allergic inflammation induces immune-mediated neuropathic pain. Anti-plexin D1 antibodies invade the dorsal root ganglia (DRG) where the blood–brain barrier (BBB) and blood–nerve barrier are absent, bind to unmyelinated small DRG neurons (primary sensory neurons), and cause neuropathic pain. Moreover, the overproduction of ET-1 (endothelin-1) in inflamed tissues induces BBB hyperpermeability and activates microglia and astroglia via the ET-1/EDNRB (endothelin receptor type B) pathway in allergic inflammation. Glial activation leads to the activation of second-order sensory neurons in the dorsal horn of the spinal cord and, ultimately, neuropathic pain.

We also found an overproduction of ET-1 in sera, alveolar epithelial cells, and skin tissues from atopic mice and elevated serum ET-1 in patients with AM. Previous studies reported increased ET-1 expression in the epidermis of atopic dermatitis patients (47) and the bronchial epithelium of asthma patients (48). Additionally, several studies reported that ET-1 attenuated BBB permeability (49). Therefore, the overproduction of ET-1 in inflamed tissues may induce BBB hyperpermeability and activate microglia and astroglia via the ET-1/EDNRB pathway in allergic inflammation. Then, glial activation might activate second-order sensory neurons in the dorsal horn of the spinal cord, causing NeP (Figure 1).

A previous study showed that the ET-1/EDNRB pathway has dual effects on the nociceptive system in response to pathological conditions (50). The ET-1/EDNRB pathway exhibited pro-nociceptive effects in inflammatory pain models (51, 52). Furthermore, because ET-1 enhances capsaicin-induced release of substance P and CGRP, as nociceptive mediators, from isolated sensory neurons without EDNRB expression, ET-1 induced pro-nociceptive effects independently of EDNRB (53). In contrast, the ET-1/EDNRB pathway exerted anti-nociceptive effects in a subcutaneous hindpaw ET-1 injection model (54) and a bone cancer model (55). In our atopic mice, the ET-1/EDNRB pathway exhibited pro-nociceptive effects. Although EDNRA is normally expressed in small DRG neurons while EDNRB is expressed in satellite glial cells and myelinating Schwann cells surrounding axons (56) (Supplementary Table 1), we have not investigated the PNS expression of EDNRA and EDNRB in our atopic mice. Further studies are required to achieve a deeper understanding of the nociceptive effects of ET-1 in allergic inflammation.

### Mechanism of Anti-plexin D1 Antibody Production in Allergic Inflammation

Although NeP patients with anti-plexin D1 antibodies have various underlying neurological diseases, they have common coexisting comorbidities, mainly allergic diseases (37), that enhance the production of autoantibodies (28). Therefore, the production of anti-plexin D1 antibodies is considered to be associated with allergic inflammation. Interestingly, the anti-plexin D1 IgG main subclass was IgG2, which predominantly recognizes carbohydrate epitopes (57). Plexin D1 is heavily glycosylated, especially at the extracellular IPT/TIG3 domain, which is the same region that immunoprecipitates as identified by mass spectrometry (37). IgG2 is preferentially produced against polysaccharides of environmental microorganisms. AM patients frequently have high levels of IgE antibodies to mite antigens, such as *Dermatophagoides pteronyssinus* (*Dpt*) and *Dermatophagoides farinae*, which are also heavily glycosylated (6, 9). Of note, IgG2 antibodies were reported to comprise up to 50% of antibodies against *Dpt* in atopic patients with high levels of anti-*Dpt* IgE antibodies (58). Thus, allergic inflammation may facilitate anti-plexin D1 antibodies through the molecular mimicry of carbohydrates such as plexin D1 and environmental allergens, including *Dpt*. IgG2 is a low inducer of complement activation and antibody-dependent cell-mediated cytotoxicity compared with IgG1 (57, 59), which might explain the observation that anti-plexin D1 antibody-positive NeP patients, especially AM patients, experience only minor disabilities other than NeP (6).

### Action of Anti-plexin D1 Antibodies

Neurological manifestations of NeP patients with anti-plexin D1 antibodies commonly include burning pain and thermal hyperalgesia (37). These symptoms reflect C-fiber type DRG neuron impairment (60). Because anti-plexin D1 antibodies specifically bind to C-fiber DRG neurons, anti-plexin D1 antibodies might be the cause of C-fiber type DRG neuron impairment and NeP. Indeed, in our *in vitro* study, anti-plexin D1 antibodies induced membrane hyperpermeability and cellular swelling of DRG neurons independent of complement activation. Because plexin D1 regulates cytoskeleton stability through actin polymerization (61), anti-plexin D1 antibodies may induce complement-independent cytotoxicity to DRG neurons through the dysregulation of cytoskeleton stability.

## Conclusion

On the basis of the above-mentioned findings, we propose that increased humoral immunity in allergic individuals may cause anti-plexin D1 antibody production through molecular mimicry with environmental allergens (Figure 1). Anti-plexin D1 antibodies can invade the DRG where the blood–nerve barrier is absent and damage primary pain-conducting neurons, triggering NeP. In addition, allergy may induce the activation of spinal microglia and astroglia via the ET1/EDNRB pathway, which might activate second-order sensory neurons and predispose allergic individuals to NeP. Although there is no evidence of a direct interaction between the ET-1/EDNRB and semaphorin/plexin D1 pathways, activation of the ET-1/EDNRB pathway may allow anti-plexin D1 antibodies to invade the CNS via the hyperpermeable BBB. Plasma exchange can remove circulating serum ET-1 and anti-plexin D1 antibodies, and ameliorate NeP associated with allergic inflammation.

Given that the prevalence of allergic diseases has been increasing over recent decades (62), we predict that allergic inflammation-related neurological diseases will also increase. Therefore, a better understanding of the neuro-immune interactions in allergic diseases might lead to novel therapeutic approaches to treat allergy-related neurological diseases.

## Author Contributions

TF, RY, and JK: study concept and design, manuscript development, writing, and funding.

### Conflict of Interest

The authors declare that the research was conducted in the absence of any commercial or financial relationships that could be construed as a potential conflict of interest.
