# Supplementary material for: Novel Neuropathic Pain Mechanisms Associated With Allergic Inflammation
Source: Front Neurol. 2019 Dec 17;10:1337. doi: 10.3389/fneur.2019.01337 (PMC6928142; doi:10.3389/fneur.2019.01337)
Supplement: Supplementary file 1 [file Table_1.docx]

Supplementary Table 1 Expressions of ET-1/EDNRA, EDNRB and SEMA3E/Plexin D1 in immune cells and the sensory nervous system.

|  | **Expression in immune cells** | **Expression in sensory nervous system** | **Refs**. |
| --- | --- | --- | --- |
| ET-1 | Endothelial cell, fibroblast, monocyte/macrophage, mast cell, neutrophil, dendric cell | Small and large DRG neurons, spinal deep dorsal horn, astroglia, activated microglia | 63-71 |
| EDNRA | Vascular smooth muscle cell, T lymphocyte, B lymphocyte, monocyte/macrophage, neutrophil, dendric cell | Small DRG neurons, spinal superficial dorsal horn (primary afferent nerve fibers) | 46, 56, 68, 72, 73 |
| EDNRB | Endothelial cell, T lymphocyte, B lymphocyte, monocyte/macrophage, neutrophil, dendric cell | satellite glial cells in DRG, myelinated Schwann cells, astroglia, activated microglia | 46, 56, 68, 71, 72, 73 |
| SEMA3E | Macrophage, vascular smooth muscle cell, thymocyte | None | 74, 75, 76 |
| Plexin D1 | Thymocyte, endothelial cell, activated B cells, macrophages, dendric cell | C-fiber type unmyelinated small DRG neurons, spinal superficial dorsal horn (primary afferent nerve fibers), VIP-positive postganglionic parasympathetic nerve fibers | 37, 76, 77, 78, 79, 80 |

DRG = dorsal root ganglion; EDNRA = endothelin receptor type A; EDNRB = endothelin receptor type B; ET-1 = endothelin 1; Refs = references; SEMA3E = semaphoring 3E; VIP = vasoactive intestinal peptide.
